# New Orleans School of Medicine

**Published:** 1859-06

**Authors:** 


### NEW ORLEANS SCHOOL OF MEDICINE.

We have received the Annual Report and Circular of the New Orleans School of Medicine, from which we learn that the third session of the Institution has closed under the most flattering circumstances, notwithstanding the late prevalence of the yellow fever in that city, during the fall of 1858. The first class of the N. O. School of Medicine, numbered 76 with 26 graduates—the second class numbered 126 with 34 graduates, and the third and last class 164 with 36 graduates.

It is one of the best Institutions in the country, and as such we take pleasure in commending it, and expressing our desire for its continued prosperity.

